# The relationship between the tertiary lymphoid structure and immune‐infiltrating cells in gastrointestinal cancers: A systematic review and meta‐analysis

**DOI:** 10.1002/iid3.70003

**Published:** 2024-09-11

**Authors:** Aoyang Yu, Zhixiang Fan, Luyao Ma, Juanjuan Tang, Wenlou Liu, Zhengxiang Han, Hongmei Wang

**Affiliations:** ^1^ Department of Oncology The Affifiliated Hospital of Xuzhou Medical University Xuzhou Jiangsu China

**Keywords:** biomarkers, gastrointestinal cancers, immune‐infiltrating cells, meta‐analysis, tertiary lymphoid structure (TLS)

## Abstract

**Objectives:**

This study systematically evaluated the relationship between tertiary lymphoid structures (TLS) and clinical pathological features as well as immune infiltrating cells in gastrointestinal cancers.

**Methods:**

We searched Web of science, Pubmed, Embase, and Cochrane Library for studies that met the requirements as of July 1, 2023, and the odds ratio, the corresponding 95% confidence interval or mean and standard deviation, were included in the analysis.

**Findings:**

We eventually included 20 studies, involving a total of 4856 patients. TLS were found to be significantly associated with T stage, N stage, TNM stage, and tumor size. Moreover, patients with positive TLS showed significantly elevated expression of T‐cell related markers, including CD3, CD4, CD8, CD45RO; B‐cell related markers, such as CD11c and CD20; and dendritic cell‐related marker CD103. On the other hand, positive TLS correlated significantly with low expression of FOXP3 and CD68. Additionally, there was a significant positive association between TLS and overall infiltration of tumor‐infiltrating lymphocytes.

**Conclusion:**

The presence of TLS is significantly correlated with the infiltration of various immune cells in gastrointestinal cancers. To determine the ideal balance between the presence of mature TLS and appropriate immune cell infiltration, further high‐quality and multicenter clinical studies need to be conducted.

## 1 INTRODUCTION

Gastrointestinal cancer primarily includes gastric cancer, esophageal cancer, liver cancer, colorectal cancer, pancreatic cancer, and cholangiocarcinoma, among others.^1^ It accounts for 19% of all cancer cases worldwide and 22.5% of all cancer‐related deaths.^2^ Research has shown that chronic inflammation, which persists for a long period of time, is the molecular and pathological basis for the development of gastrointestinal cancer.^3^ Although chronic inflammation can create a microenvironment that favors invasive disease spread,^4^, ^5^, ^6^, ^7^ increasing evidence suggests that in certain malignant gastrointestinal cancers, the immune cell infiltration resulting from chronic tissue damage might inhibit tumor growth and potentially regulate the clinical progression of the disease.^8^, ^9^, ^10^, ^11^, ^12^ In the era of immunotherapy, therapy targeting immune‐infiltrating cells has emerged as a safe and effective immunological treatment and has gained attention. However, limited understanding of the immune microenvironment restricts its success in gastrointestinal tumors.^13^ Tertiary lymphoid structures (TLS) are ectopic lymphoid tissues that form in sites of chronic inflammation, including tumors. Structurally, TLS resembles secondary lymphoid organs and is mainly composed of B cells, T cells, dendritic cells (DC), and fibroblastic reticular cells.^14^ In various gastrointestinal cancers, TLS serves as a potential biological marker, as its presence and expression are significantly correlated with the prognosis of several tumors.^15^, ^16^, ^17^, ^18^ In tumor tissues with TLS, a unique immune cell infiltration landscape is observed. TLS can serve as a site for the local production of tumor antigen‐specific B cells and T cells, catalyzing B cell differentiation and maturation, as well as antigen presentation to T cells.^19^ However, the molecular mechanisms underlying the formation and maintenance of TLS in tissues are not yet clear, and there is ongoing controversy regarding the relationship between various immune cells and TLS in real‐world studies.^16^, ^20^, ^21^, ^22^ This study systematically evaluates the relationship between TLS and clinicopathological features, as well as immune cell infiltration in gastrointestinal tumors, providing new insights for understanding the mechanisms of TLS formation and further development of immune cell therapies.

## 2 METHODS

### 2.1 Search strategy

According to PRISMA guidelines, we systematically searched literature sources from the following databases: Pubmed, Embase, Web of Science and the Cochrane library, with the language restricted to English. The last search was updated on July 1, 2023. Search terms were as follows: (Tertiary Lymphoid Structures OR Lymphoid Structure, Tertiary OR Lymphoid Structures, Tertiary OR Tertiary Lymphoid Structure OR Ectopic Lymphoid‐Like Structures OR Lymphoid‐Like Structure, Ectopic OR Ectopic Lymphoid Tissues). three authors (Menghan Cao, Kaile Zhang and Yule Yang) independently executed the retrieval according to the standardized process. Any disagreement between the three authors was resolved through discussion.

### 2.2 Inclusion and exclusion criteria

The studies that meet the criteria include the following: (1) gastrointestinal cancers confirmed by pathological diagnosis; (2) detection of TLS expression levels in human tumor tissues; (3) providing at least one example that demonstrates the relationship between TLS and immune cells or related molecular markers, using measures such as odds ratio (OR), 95% confidence interval (CI), or mean ± standard deviation (*SD*). Alternatively, providing at least one visual representation such as a bar chart, box plot, or violin plot, illustrating the relationship between TLS and immune cells or related molecular markers; (4) providing the methods for TLS detection and evaluation.

Exclusion criteria: (1) other types of cancers; (2) comment, letter, edit, reviews and meta‐analysis; (3) no insufficient data; (4) no available information of tumor‐infiltrating immune cells; (5) repeated research on the same sample queue.

### 2.3 Data extraction and quality assessment

The data extracted from the included studies include the following: year of publication, region, sample size, cancer type, TLS cut‐off criteria, study period, and information on the immunological cells and cluster of differentiation used to evaluate the relationship between TLS and immune cell markers. Assessment of the relationship is typically done using metrics such as ORs, 95% CIs, or mean ± *SD*. In cases where OR, 95% CI, or mean ± *SD* are not provided in the original studies, the Engauge Digitizer software version 4.1 is utilized to evaluate graphs such as bar charts, box plots, or violin plots. By using a conversion template developed by Drahota et al., the extracted values such as mean, *SD*, quartiles, or range can then be converted to mean ± *SD* format.^23^ Quality assessment was performed using the Newcastle–Ottawa quality assessment scale (NOS). NOS criteria scores range from 0 (*lowest*) to 9 (*highest*), and a NOS score ≥5 is considered a high‐quality study. Two reviewers (Luyao Ma and Xinran Zhang) independently assessed the quality of the eligible studies and extracted the data, and any disagreement was resolved through discussion with the third (Xiao Ma).^24^

### 2.4 Statistical analysis

Statistical analysis was performed using Stata 15.0, which computed the correlation between TLS and overall infiltration of tumor‐infiltrating lymphocytes (TIL), as well as the expression levels of CD3, CD4, CD8, CD11c, CD20, CD45RO, CD68, CD103, and FoxP3. A significance level of *p* < .05 and an *I* ^2^ value of >50% indicated high heterogeneity, therefore a random‐effects model was applied. Otherwise, a fixed‐effects model was used. Egger's and Begg's tests were employed to evaluate publication bias. If significant publication bias was present, a trim‐and‐fill method was used to adjust the results.^25^ Furthermore, a sensitivity analysis was performed using the one‐by‐one exclusion method. Results with *p* < .05 were considered to be statistically significant.

## 3 RESULTS

### 3.1 Characteristics

**Figure 1:**
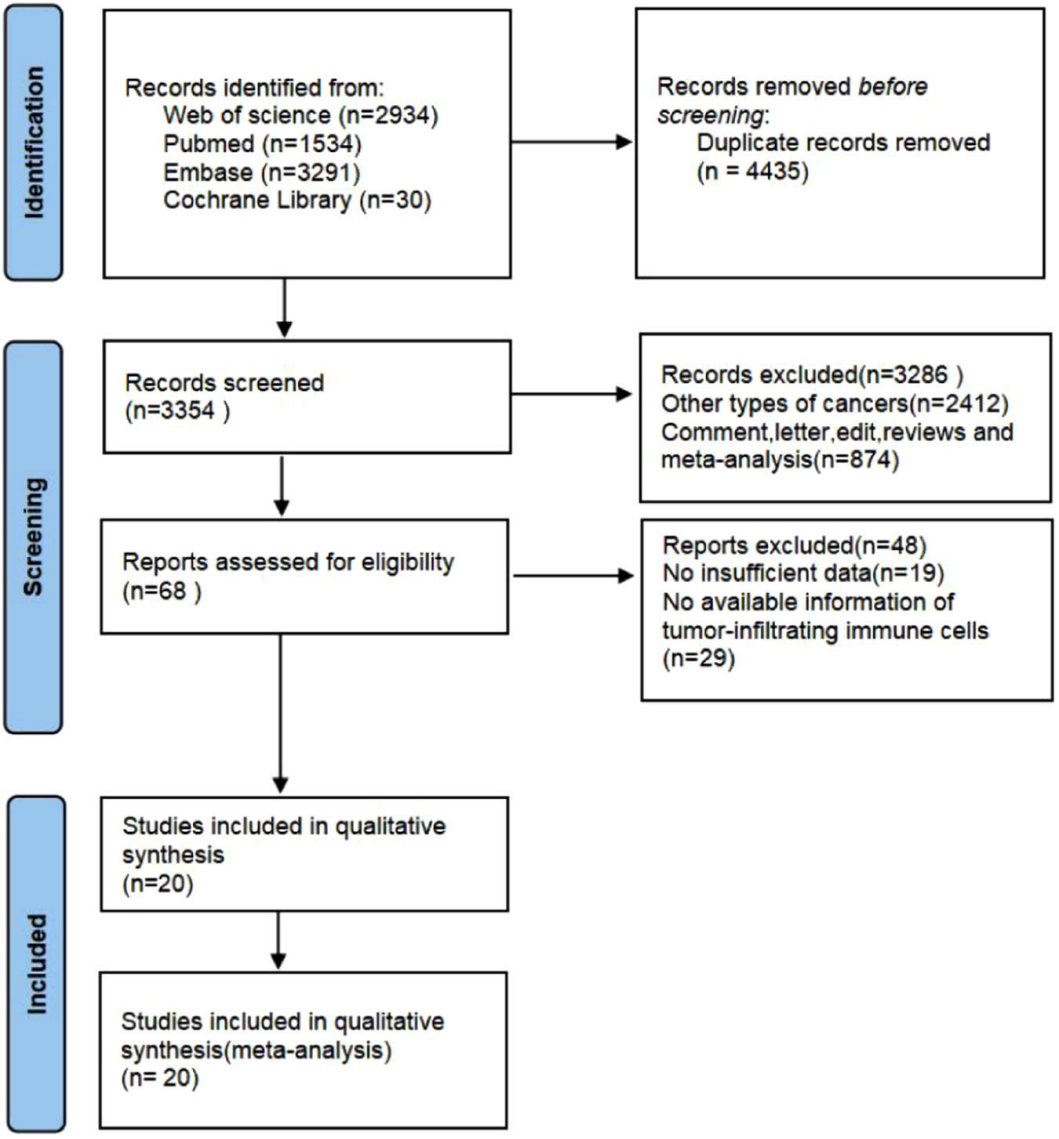
Flow diagram of literature retrieval strategy.

After an initial search, we identified and removed 4435 duplicate articles. Subsequently, we carefully evaluated the titles and abstracts of the remaining articles and excluded 3286 articles that were not relevant to our study. We then assessed the full texts of the remaining 64 articles and finally included 20 studies in our analysis.^20^, ^26^, ^27^, ^28^, ^29^, ^30^, ^31^, ^32^, ^33^, ^34^, ^35^, ^36^, ^37^ These 20 studies encompassed a total of 4856 patients, with 6 studies focusing on gastric cancer (GC), 5 on pancreatic cancer (PC), 4 on hepatocellular carcinoma (HCC), 3 on colorectal cancer (CRC), and 2 on esophageal cancer (EC). Among these studies, 12 were conducted in China, 4 in Japan, and the remaining 4 in European countries. Regarding the evaluation of TLS, the included studies employed different cut‐off criteria to determine their presence, density, and maturity degree. TLS is divided into TLS‐high and TLS‐low based on different cut‐off criteria. The immune cells and molecular markers investigated in these articles included TIL, CD3, CD4, CD8, CD11c, CD20, CD21, CD31, CD45RO, CD57, CD68, CD69, CD103, CD138, follicular dendritic cell (FDC), and FoxP3. To assess the quality of the included studies, we used the NOS scoring system. The NOS scores of the included studies ranged from 5 to 8, indicating that the included studies had relatively high quality (Table 1). The PRISMA flowchart depicting the entire selection process can be seen in the provided (Figure 1).

**Table 1:** Characteristics of included studies.

Study	Year	Cancer types	Reigon	Patients	Cut‐off criteria	Study period	Immunol cells and cluster of differentiation	ROC
Cheng et al.^26^	2021	GC	China	846	Presence	2001/1–2013/12	TIL	7
Yin et al.^27^	2022	GC	China	148	Presence	2015/1–2019/12	CD4, CD8, CD20, CD23, FDC, FoxP3	7
Mori et al.^28^	2021	GC	Japan	261	Density	2014–2017	CD8, CD20, CD103	8
He et al.^29^	2020	GC	China	914	Density	2002–2008	CD21	7
Mori et al.^30^	2022	GC	Japan	19	Density	2017–2020	CD20, CD103	5
Ji et al.^31^	2022	GC	China	118	Density	2009–2014	CD3, CD8, CD20, CD68	8
Zhan et al.^38^	2022	CRC	China	203	Density	2014/1–2017/7	CD3, CD8, CD31	6
Schweiger et al.^32^	2016	CRC	Austria	57	Presence	2009/4–2014/6	CD3, CD8, CD45RO, FoxP3	6
Karjula et al.^33^	2023	CRC	Finland	63	Density, Presence	2000–2020	CD3, CD8	7
Meylan et al.^34^	2020	HCC	France	41	Presence	2005–2015	CD3, CD4, CD8, CD20, CD163	6
Li et al.^39^	2021	HCC	China	240	Presence, Density	2019–2013	CD3, CD8, CD20, CD57, CD68, FoxP3	8
Li et al.^35^	2020	HCC	China	303	Presence	2009/3–2013/8	CD3, CD8, CD20, CD68, FoxP3	7
Zhang et al.^36^	2020	HCC	China	170	Presence	2016–2018	TIL	8
Hayashi et al.^37^	2023	EC	Japan	316	Density, Maturity degree	2001/1–2017/12	TIL, CD138	8
Li et al.^20^	2022	EC	China	185	Density, Presence	2017/1–2018/12	CD4, CD8, CD11c, CD20, CD45, CD68	7
Zhang et al.^16^	2020	PC	China	182	Presence	2016–2018	CD4, CD8, CD11c, CD20, CD45, CD68	7
Zou et al.^21^	2023	PC	China	380	Presence	2013–2019	CD4, CD8, CD20, CD30, CD69	7
Zou X et al.^21^	2023	PC	China	136	Presence	2013–2029	CD4, CD8, CD20, CD69	8
Ahmed et al.^22^	2022	PC	Germany	55	Presence	2007/3–2011/7	CD8, CD20	6
Tanaka et al.^40^	2023	PC	Japan	219	Presence	2018/4–2017/3	CD4, CD8, CD20, CD45, CD68, FoxP3	7

Abbreviations: CRC, colorectal cancer; EC, esophageal cancer; GC, gastric cancer; HCC, hepatocellular carcinoma; PC, pancreatic cancer.

### 3.2 The relationship between TLS and clinical pathological features

**Figure 2:**
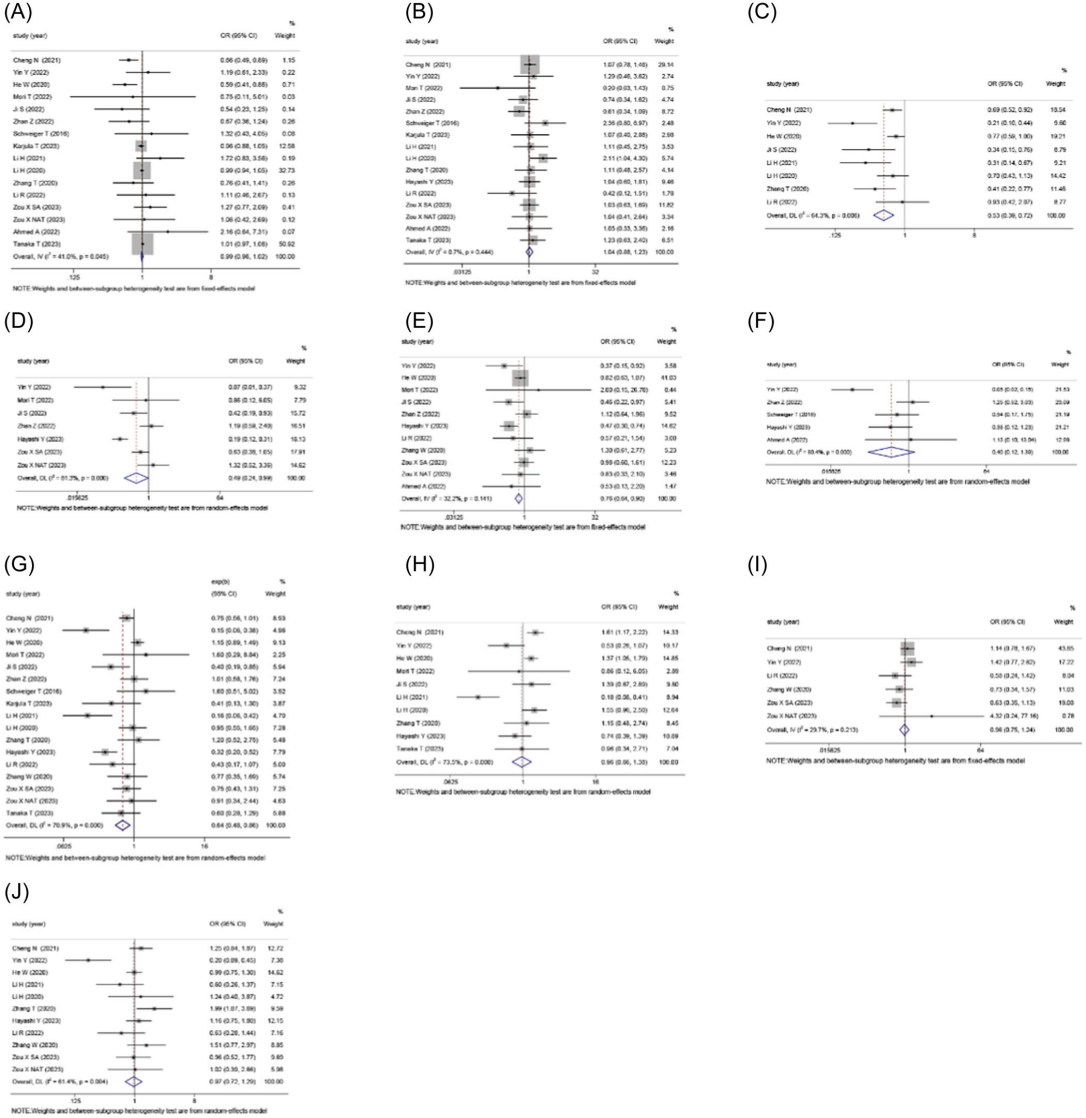
Forest plot of the OR for the association between the TLS and clinicopathological features in patients with gastrointestinal cancers. (A) age; (B) sex; (C) tumor size; (D) T stage; (E) N stage; (F) M stage; (G) TNM stage; (H) lymph vascular invasion; (I) perineural invasion (J) histologic differentiation. OR, odds ratio; TLS, tertiary lymphoid structure.

The study included 18 studies that investigated the relationship between TLS and clinical pathological characteristics of digestive tract tumors. The results showed that TLS‐high was significantly associated with tumor size (OR: 0.532, 95% CI: 0.392–0.721, *p* < .001) (Figure 2C), T stage (OR: 0.491, 95% CI: 0.224–0.989, *p* = .047) (Figure 2D), N stage (OR: 0.758, 95% CI: 0.638–0.901, *p* = .002) (Figure 2E) and TNM stage (OR: 0.639, 95% CI: 0.477–0.856, *p* = .003) (Figure 2G). However, TLS‐high was not significantly associated with age (OR: 0.989, 95% CI: 0.958–1.021, *p* = .047) (Figure 2A), sex (OR: 1.039, 95% CI: 0.876–1.232, *p* = .663) (Figure 2B), M stage (OR: 0.401, 95% CI: 0.116–1.389, *p* = .047) (Figure 2F), lymph vascular invasion (OR: 0.967, 95% CI: 0.722–1.295, *p* = .822) (Figure 2H), perineural invasion (OR: 0.963, 95% CI: 0.747–1.242, *p* = .772) (Figure 2I), and histologic differentiation (OR: 0.957, 95% CI: 0.664–1.381, *p* = .816) (Figure 2J).

### 3.3 The relationship between TLS and immune‐infiltrating cells

**Figure 3:**
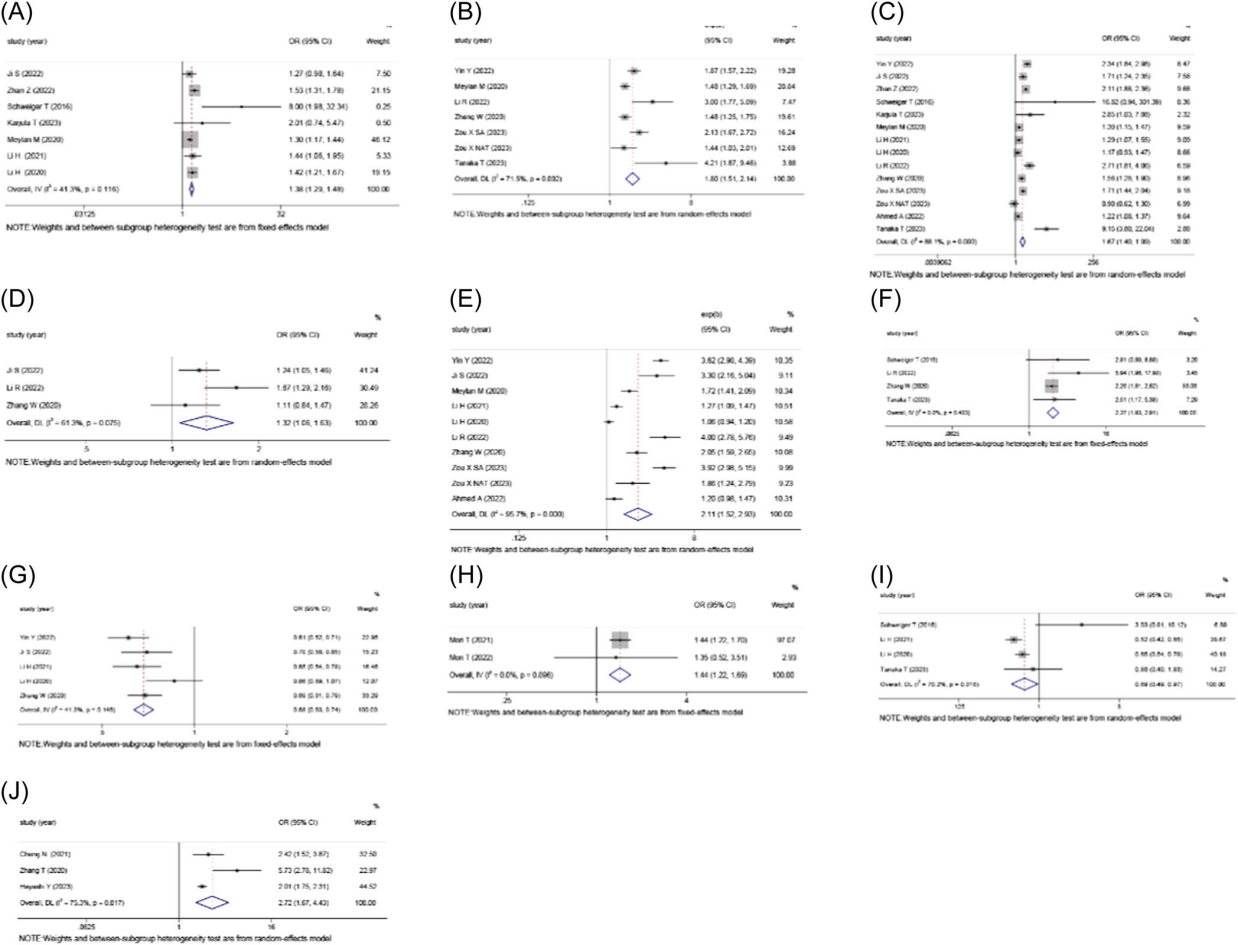
Forest plot of the OR for the association between the TLS and immune‐infiltrating cells in patients with gastrointestinal cancers. (A) CD3; (B) CD4; (C) CD8; (D) CD11c; (E) CD20; (F) CD45RO; (G) CD68; (H) CD103; (I) Foxp3 (J) TIL. OR, odds ratio; TIL, tumor‐infiltrating lymphocyte; TLS, tertiary lymphoid structure.

Based on the findings of the included 20 studies, it was observed that TLS was significantly correlated with various immunological cells and cluster of differentiation markers. TLS‐high was strongly associated with the expression of T‐cell associated molecules, such as CD3 (OR: 1.383, 95% CI: 1.289–1.484, *p* < .001) (Figure 3A), CD4 (OR: 1.797, 95% CI: 1.510–2.139, *p* < .001) (Figure 3B), CD8 (OR: 1.668, 95% CI: 1.398–1.991, *p* < .001) (Figure 3C), and CD45RO (OR: 2.371, 95% CI: 1.930–2.912, *p* < .001) (Figure 3F). Conversely, TLS‐high was negatively associated with the expression of FOXP3 (OR: 0.690, 95% CI: 0.489–0.793, *p* = .034) (Figure 3I). Moreover, TLS‐high was significantly correlated with the elevated expression of B‐cell associated markers, including CD11c (OR: 1.316, 95% CI: 1.062–1.630, *p* = .012) (Figure 3D) and CD20 (OR: 2.108, 95% CI: 1.517–2.931, *p* < .001) (Figure 3E). Conversely, TLS‐high showed a significant negative correlation with the expression of macrophage‐associated marker CD68 (OR: 0.684, 95% CI: 0.634–0.737, *p* < .001) (Figure 3G). Additionally, TLS‐high was positively correlated with the expression of DC‐associated marker CD103 (OR: 1.437, 95% CI: 1.221–1.692, *p* < .001) (Figure 3H). Last, TLS‐high was strongly associated with high expression of TILs (OR: 2.717, 95% CI: 1.666–4.432, *p* < .001) (Figure 3J).

### 3.4 Sensitivity analysis and publication bias

Due to the limited availability of literature regarding the relationship between TLS and CD11C, CD45RO, CD103, FOXP3, and TIL, there is an inevitable instability in the sensitivity analysis results and publication bias. In this stage, we primarily conducted sensitivity analysis and publication bias analysis by including five or more studies.

**Figure 4:**
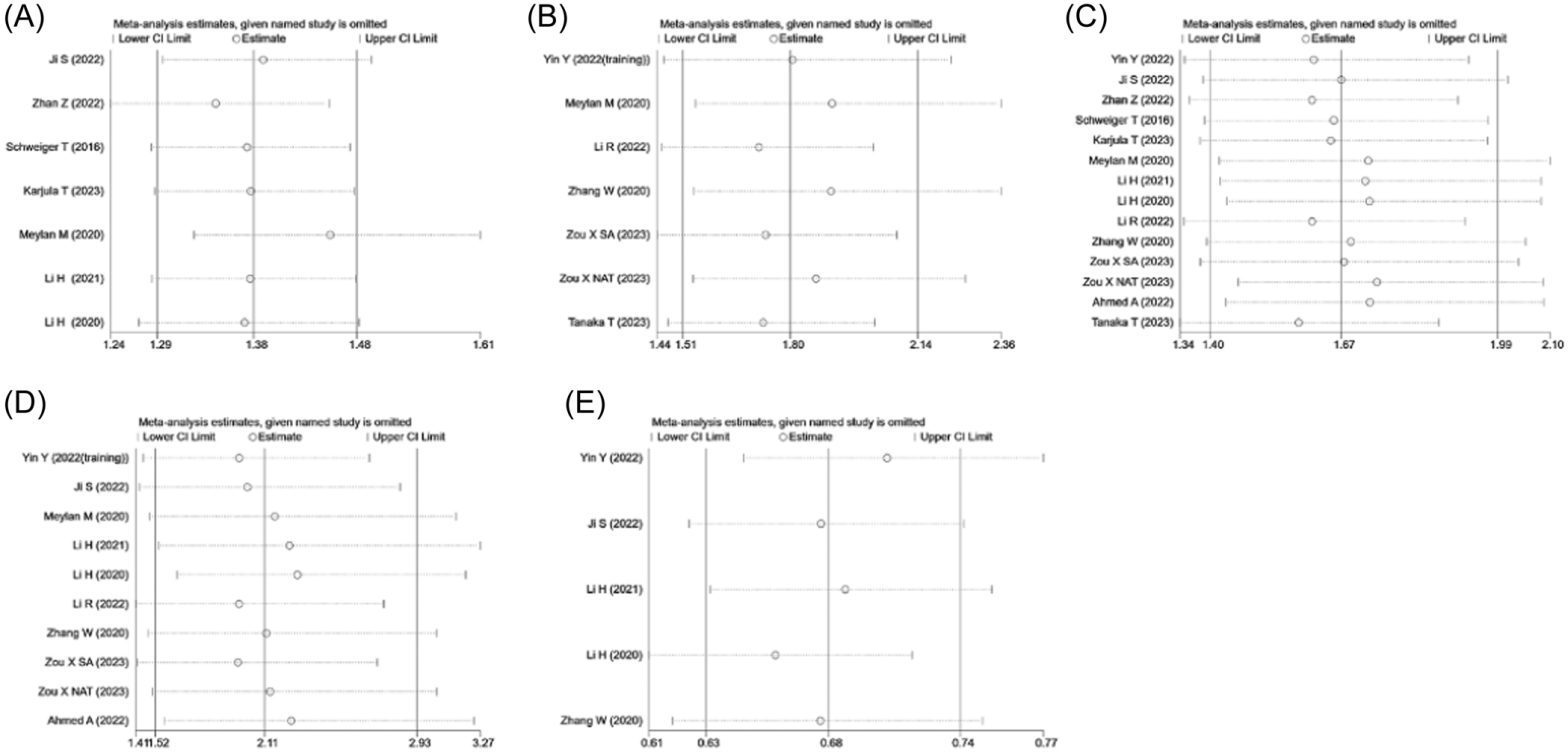
Sensitivity analysis. (A) CD3; (B) CD4; (C) CD8; (D) CD20; (E) CD68.

To assess sensitivity, we conducted a statistical analysis using the leave‐one‐out method. After systematically excluding each individual study, the overall ORs for TLS with respect to CD3, CD4, CD8, CD20, and CD68 did not show any significant changes, indicating the stability of our study results(Figure 4). Next, we evaluated publication bias of the included studies using Begg's test and Egger's test. The results for CD4 (Begg's test: *p* = .016, Egger's test: *p* = .066) (Figures S1b and S2b) and CD20 (Begg's test: *p* = .153, Egger's test: *p* = .003) (Figures S1d and S2d) indicated the presence of publication bias. However, CD3 (Begg's test: *p* = .230, Egger's test: *p* = .084) (Figures S1a and S2a), CD8 (Begg's test: *p* = .228, Egger's test: *p* = .283) (Figures S1c and S2c), and CD68 (Begg's test: *p* = .221, Egger's test: *p* = .409) (Figures S1e and S2e) did not show any publication bias. Subsequently, we used the trim and fill method to assess the missing studies related to CD4 and CD20. In the CD4‐related studies, after 5 rounds of iteration, 2 missing studies were imputed, and the merged results remained statistically nonsignificant (OR: 1.658, 95% CI: 1.375–1.999, *p* < .001) (Figure S3a). In the CD20‐related studies, no missing studies were imputed, indicating the stability of our results (Figure S3b).

## 4 DISCUSSION

Although previous studies have confirmed that TLS and immune infiltrating cells are potential biological markers for the prognosis of gastrointestinal cancer, the relationship between TLS and immune infiltrating cells remains controversial.^41^, ^42^ Recent research suggests that the formation of TLS is mediated by some proinflammatory cytokines and TNF receptor family members, involving fibroblasts, perivascular smooth muscle cells, and stromal cells.^43^ However, in gastrointestinal cancer, a large number of patients have low tumor mutation burden and lack immune cell infiltration, which is considered to make the tumor microenvironment “cold” and lead to poor response to emerging therapies targeting the tumor immune microenvironment, such as immunotherapy.^44^, ^45^ Hooren et al.^46^ found the formation of TLS during the process of transforming the immune microenvironment of solid tumors from “cold” to “hot” using a CD40 agonist, and TLS‐high was associated with increased infiltration of T cells. We speculate that an increase in appropriate immune cell infiltration may be associated with the formation of TLS, and the presence of TLS can also promote the infiltration of local immune cells. Therefore, we systematically evaluated the relationship between TLS and immune infiltrating cells in gastrointestinal cancer, aiming to identify an immune microenvironment suitable for the presence of TLS. Our research has found a significant correlation between high expression levels of T cell‐related molecular markers CD3, CD4, CD8, CD45RO, B cell‐related molecular marker CD20, DC‐related molecular marker CD103, and B cell and DC‐related molecular marker CD11c with TLS. Thompson et al. found that TLS may promote an antitumor immune response by inducing T cells to produce an immune response that is independent of conventional lymphoid organ initiation.^47^ Goc et al.^48^ reported that T cells in TLS are mainly composed of CD4^+^ T cells and can enhance the recruitment, activation, and effector functions of CD8^+^ T cells in various ways. These results suggest that TLS may play an important role in initiating TIL during the local immune response production process and contribute to the formation of a favorable tumor immune environment for survival. In mature TLS, a functional germinal center can be observed, consisting of a large number of clustered B cells and follicular DCs. This germinal center is a critical site for the immune functions of TLS.^14^ Fridman et al.^49^ reported that in mature TLS, B cells undergo proliferation and generate plasma cells to exert antitumor effects. Some studies have shown that stimulating B cells through tumor vaccines, neoadjuvant chemotherapy, and targeted radiotherapy can promote the formation of TLS, thereby improving the potential outcome of immunotherapy.^50^, ^51^, ^52^ Thompson et al.‘s research findings suggest that mature DCs in TLS can present tumor‐associated antigens, directly promoting the sustained production of specific T cells within the tumor. Therefore, TLS can induce local immune responses that are better adapted to changes in tumor‐associated antigen expression during tumor progression.^48^

Based on the included studies, the research has shown a significant correlation between TLS‐high and low expression of FOXP3 and CD68. Despite the fact that FOXP3^+^ T cells, as regulatory T cells, can inhibit effective immune responses against cancer cells, recent studies have suggested that FOXP3^+^ T cells may be associated with favorable prognosis.^53^, ^54^ De Leeuw et al.^55^ conducted a comprehensive review on the role of FOXP3^+^ T cells in cancer and found that the prognostic effect of FOXP3^+^ T cells is heavily influenced by the tumor site. It is associated with poor prognosis in hepatocellular carcinoma and generally good prognosis in colorectal cancer, while inconsistent or insufficient research has been conducted on other types of cancer.^55^ The studies we included in our research investigating the relationship between TLS and FOXP3 expression levels consist of one study on colorectal cancer, two studies on liver cancer, and one study on pancreatic cancer. There is significant heterogeneity among these studies. The included studies showed a significant correlation between high expression of TLS and FOXP3 in pancreatic cancer patients. On the other hand, low expression of TLS and FOXP3 was significantly associated in gastric cancer and liver cancer patients. It is worth noting that a larger proportion of liver cancer patients included in the study had Lymphocyte‐rich hepatocellular carcinoma (LR‐HCC). LR‐HCC itself is quite rare, and it has not been fully characterized in terms of clinical pathology and molecular studies. This lack of comprehensive understanding in LR‐HCC could potentially lead to biases in our conclusions. In the studies included in our research on the relationship between TLS and FOXP3 expression levels, there were 1 colorectal cancer study, 2 hepatocellular carcinoma studies, and 1 pancreatic cancer study, all of which showed significant heterogeneity. According to Zhang et al.,^56^ high levels of CD68 are associated with many immune cells in the tumor microenvironment, such as monocytes, B cells, CD4^+^ and CD8^+^T cells, DCs, macrophages, and neutrophils. We speculate that the infiltration of CD68^+^ macrophages may affect the activation process of various immune cells in TLS, thereby inhibiting the formation of TLS.

Despite our main findings being reliable, there are some limitations to our meta‐analysis. First, there is a scarcity of literature included in our investigation of the relationship between TLS and the immune cell markers CD11c, CD45RO, CD103, FOXP3, and TIL. As a result, we encountered unavoidable instability in our sensitivity analysis and publication bias. Secondly, significant heterogeneity was noted in studies related to CD4, CD8, CD20, and other relevant markers. A study by Zhou et al.^21^ included two independent research cohorts that employed the same methods for detecting the aforementioned immune cell markers and TLS. However, these two cohorts still exhibited significant differences in their results.^21^ These findings suggest that there may be inherent errors in the detection of immune cell markers and caution is needed when interpreting the relationship between TLS and immune cells in gastrointestinal cancer.

In summary, the high expression of T‐cell‐related molecular markers CD3, CD4, CD8, CD45RO, B‐cell‐related molecular marker CD20, DC‐related molecular marker CD103, and B‐cell and DC‐related molecular marker CD11c is significantly correlated with TLS and gastrointestinal tumors. On the other hand, low expression of FOXP3 and CD68 is also significantly associated with TLS and gastrointestinal cancer. Additionally, low expression of FOXP3 and CD68 is also significantly correlated with TLS. However, further high‐quality and multicenter clinical studies are needed to elucidate the relationship between TLS and immune cells in gastrointestinal cancer and other solid tumors. This will help understand the balance between the presence of mature TLS and appropriate immune cell infiltration.

## AUTHOR CONTRIBUTIONS

*Conceptualization*: Aoyang Yu, Luyao Ma, and Zhengxiang Han. *Methodology*: Zhixiang Fan. *Formal analysis and investigation*: Aoyang Yu, Zhengxiang Han and Hongmei Wang. *Writing‐original draft preparation*: Aoyang Yu and Juanjuan Tang; *Writing‐review and editing*: Wenlou Liu and Zhengxiang Han. *Funding acquisition*: Zhengxiang Han and Hongmei Wang. *Resources*: Aoyang Yu and Luyao Ma. *Supervision*: Wenlou Liu, Zhengxiang Han and Hongmei Wang.

## CONFLICT OF INTEREST STATEMENT

The authors declare no conflicts of interest.

## Supporting information

Supporting information.
