# Supplementary material for: The relationship between the tertiary lymphoid structure and immune‐infiltrating cells in gastrointestinal cancers: A systematic review and meta‐analysis
Source: Immun Inflamm Dis. 2024 Sep 11;12(9):e70003. doi: 10.1002/iid3.70003 (PMC11389262; doi:10.1002/iid3.70003)
Supplement: Supplementary file 1 — Supporting information. [file IID3-12-e70003-s001.docx]

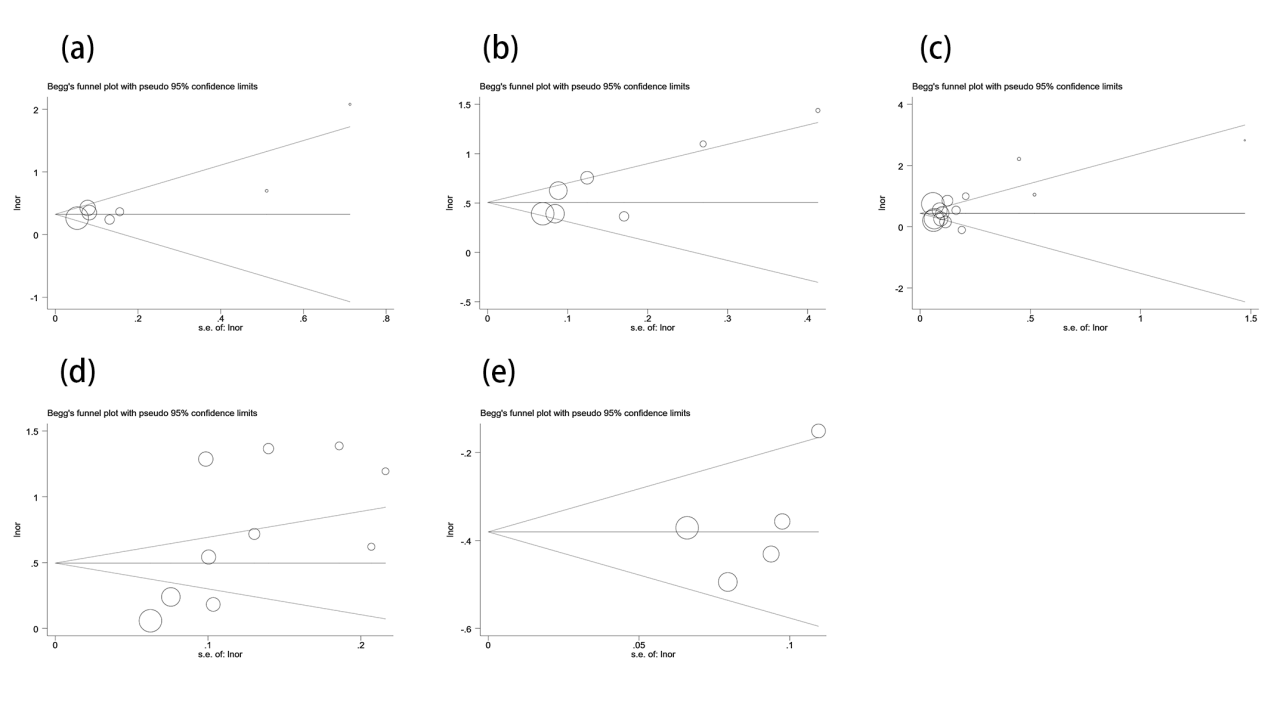


Fig S1. Begg’s funnel plots for detecting publication bias. a CD3; b CD4; c CD8; d CD20; e CD68.


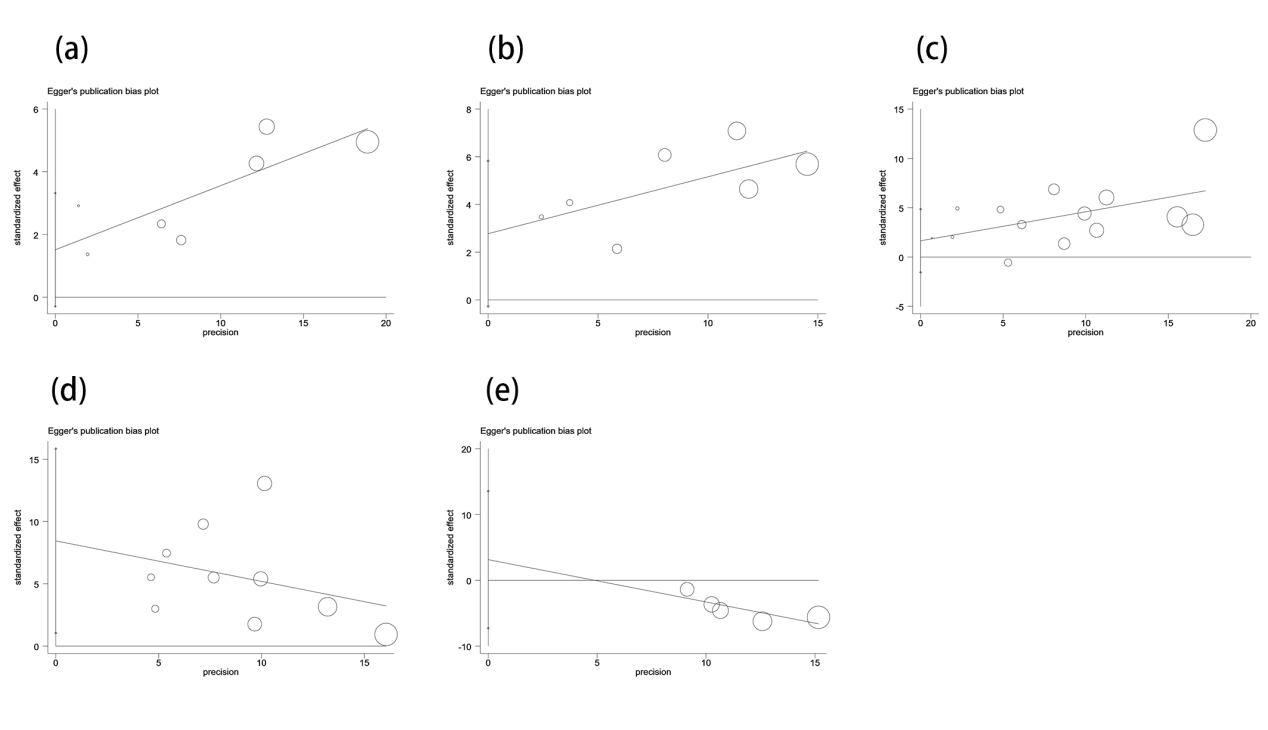
 Fig S2. Egger’s funnel plots for detecting publication bias. a CD3; b CD4; c CD8; d CD20; e CD68.


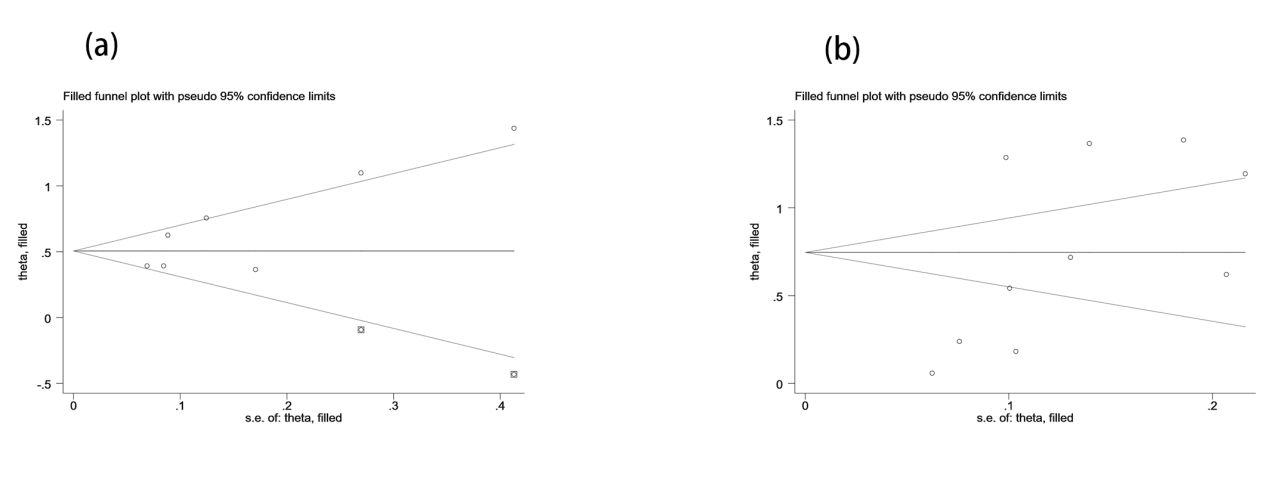
 Fig S3. Trim and fill method’s funnel plots for detecting publication bias. a CD4; b CD20.
